# Support for infant and young child feeding and care in emergencies: experiences from Australia and eleven other high-income countries

**DOI:** 10.3389/fnut.2026.1940793

**Published:** 2026-09-17

**Authors:** Karleen D. Gribble, Alexandra Shanks, Michelle Hamrosi

**Affiliations:** 1 School of Nursing and Midwifery, Western Sydney University, Parramatta, NSW, Australia; 2 Australian Breastfeeding Association, Melbourne, VIC, Australia; 3 School of Medicine, Australian National University, Canberra, ACT, Australia

**Keywords:** breastfeeding, disasters, formula feeding, infants, IYCF-E, mothers, emergency planning

## Abstract

**Introduction:**

In 2010, the World Health Assembly urged all member states, including those of high income, to implement disaster plans in line with the Operational Guidance for Infant and Young Child Feeding in Emergencies (OG-IFE). However, analyses of infant and young child feeding in emergencies (IYCF-E) planning across Europe, the United States of America (USA) and Australia have indicated a lack of such planning. This study aimed to identify challenges, lessons and solutions in infant and young child feeding and care in disasters in high-income countries to inform improved emergency planning in Australia and other high-resource settings.

**Method:**

Semi-structured interviews were conducted with 39 emergency responders from Australia and 20 emergency responders from Canada, Croatia, Iceland, Ireland, Italy, Japan, New Zealand, Poland, the United Kingdom, Ukraine, and the USA. Data was collected on the challenges of providing emergency response to infants and young children and their caregivers, as well as successful interventions and facilitators of intervention uptake. Interviews were recorded, transcribed and then analysed using reflexive thematic analysis. Through the analysis process, the OG-IFE and the World Health Organization’s nurturing care in emergencies brief assisted understanding regarding challenges, gaps and response appropriateness.

**Results:**

Challenges to support infants and young children in emergencies were consistent between countries and involved lack of prioritisation, planning, and data collection regarding infants and young children; lack of planning, knowledge and appropriate support for IYCF-E; and the donation of infant formula. Interventions to support mothers and caregivers of infants and young children were more variable between countries and included making support needs and gaps visible, providing tailored evacuation centre support, implementation of IYCF-E interventions, and supporting responsive caregiving. Intervention uptake was facilitated via training staff and volunteers, support of community members, involvement of government agencies and UNICEF, and collaboration between health agencies and emergency organisations. Family resilience was promoted at individual and community levels.

**Conclusion:**

Infants and young children are vulnerable in emergencies globally. This research has identified challenges and a range of strategies to strengthen disaster planning in high-income countries, including the appropriate development and implementation of IYCF-E plans aligned with the OG-IFE.

## 1 Introduction

Infants and young children are vulnerable in emergencies due to their developmental immaturity; specialised nutritional needs; susceptibility to heat, dehydration and cold; and total dependence on others (1). Recognising vulnerability specifically related to feeding, in 2010 the World Health Assembly urged all member states to implement infant and young child feeding in emergencies (IYCF-E) plans in line with the Operational Guidance for Infant and Young Child Feeding in Emergencies (OG-IFE) (2). However, a 2016 survey of 84 countries undertaken as part of the World Breastfeeding Trends Initiative (WBTi) showed IYCF-E was the most overlooked area of breastfeeding policy globally (3). Similarly, a 2020 WBTi analysis involving 18 European countries and a 2025 United States of America (USA) WBTi analysis found IYCF-E was, respectively, ‘seriously neglected’ (4) and ‘poorly integrated’ (5) in emergency preparedness.

Australia regularly experiences natural disasters that threaten lives and property, but children have been largely missing from emergency planning. An audit conducted by Save the Children Australia in 2016 concluded children had been overlooked in disaster plans with their needs ‘not routinely and systematically considered’ (6, p. 9). Research conducted in 2018 as a part of a WBTi assessment found that, while Australian emergency guidance identified children as a vulnerable group in need of targeted support, there was ‘a dearth of planning at all levels of government for the needs of infants and young children’ (7, p. 7), including with respect to IYCF-E. In 2019, Australian governments took a step towards improved planning with the publication of the Australian National Breastfeeding Strategy, which included breastfeeding support in emergencies and development of an IYCF-E policy as priority actions (8). However, the unprecedented 2019–20 Black Summer Bushfires immediately followed and lack of emergency planning for infants and young children was evident and impactful. For example, the evacuation of a coastal town by the Navy saw adults, older children and pets rescued, while pregnant women and those with infants and young children were left behind in heavy smoke because their safe transfer and carriage on the ship had not been adequately planned (9).

Following this disaster and in recognition of lack of preparedness, the Australian Government funded the Australian Breastfeeding Association in 2022 to undertake the Community Protection for Infants and Young Children in Bushfire Emergencies Project (ABA Bushfire Project). The goals of the ABA Bushfire Project were to:

- increase understanding of the needs of infants and young children and their mothers and other caregivers in emergencies;
- support individuals and organisations to develop or adjust their emergency plans to better support families with very young children during and after emergencies.

The first stage of the ABA Bushfire Project was the Babies and Young Children in the Black Summer (BiBS) Study, which commenced in August 2022. This paper reports on aspects of the BiBS Study that investigated emergency responder experiences, policy and practice to support families with infants and young children in high-income countries, with a focus on IYCF-E. To our knowledge, this research is the first to identify and describe commonalities in challenges, lessons learned and effective solutions in emergency response for infants and young children in high-income countries. Through this multi-national approach, it aims to advance knowledge on IYCF-E and assist to improve emergency planning by providing insight into strategies suitable for use in high-resource settings.

## 2 Method

Semi-structured interviews were conducted with individuals who had been involved in emergency planning or response in Australia and other high-income countries. Conduct of the research, including recruitment and data analysis, was informed by the experiences of the first and third authors; respectively a long-time researcher and policy maker in IYCF-E active at a global level and a general practitioner physician who lives and works in an emergency-prone region of Australia. Australian study participants were either known to the first or third author or were recruited through advertisement on social media or snowball sampling. Non-Australian study participants were either known to the first author or identified by snowball sampling. Recruitment of Australian participants focused on obtaining a range of experience across emergency response (not necessarily specific to infants and young children). Recruitment of participants outside Australia targeted those whose work was primarily or largely related to infants and young children, particularly IYCF-E, and aimed to include participants from diverse countries, especially those which experience major disasters. The differential process and focus in recruitment of Australian versus other study participants was partly a function of the research being a part of a larger project focussed on improving Australian disaster response and being based in Australia. Due to the exploratory nature of the research and a tight timeframe for data collection (again related to the research being a part of a larger time-limited project), it was not intended that data saturation would be reached. Rather, targeted recruitment of participants with a breadth of experiences was undertaken within the time available to increase understanding on how to practically support caregivers of infants and young children in emergencies in high-resource settings.

Individuals invited to participate in the study were provided with a participant information sheet outlining the nature of the research and advised that there was no obligation to be involved. Written consent was obtained before interviews, and ethical approval was granted by the Western Sydney University Human Research Ethics Committee (number H15019). Semi-structured interviews were conducted in English with separate interview guides for Australian and international study participants. All interviewees were asked about their emergency experiences related to infants and young children, emergency policy and practices to support the parents and caregivers of infants and young children and what could be done to better support these families. Additionally, Australian interviewees were asked about barriers to providing good support, while international interviewees were asked to describe any effective emergency planning and responses they were aware of.

Interviews were undertaken via Zoom^®^ from September 2022 to March 2023 and lasted for approximately 1 hour. Transcribed and deidentified interviews were uploaded to NVivo® and a reflexive thematic analysis carried out by the first author involving familiarisation with the data including note taking, initial deductive coding, and thematic generation (10, 11). Data analysis focused on identifying common challenges across countries, along with innovations and promising solutions to support the mothers and other caregivers of infants and young children in emergencies. Through the analysis process, the OG-IFE and the World Health Organization’s (WHO) nurturing care in emergencies brief assisted understanding regarding challenges, gaps and appropriateness of responses (12).

## 3 Results

Thirty-nine Australian emergency responders representing a breadth of disaster response roles were interviewed as shown in Table 1. Twenty emergency responders from Canada, Croatia, Iceland, Ireland, Italy, Japan, New Zealand, Poland, the United Kingdom (UK), Ukraine, and the USA were interviewed as shown in Table 2. Six of the 39 Australian study participants and six of the 20 international study participants were individuals known to the researchers. Themes and subthemes identified are summarised in Table 3. Quotations were selected to illustrate themes rather than to achieve equal representation across countries. Differences in the number of quotations attributed to individual countries reflects variations in participant numbers, the richness of the data collected, and the relevance of participants’ responses to the study aims.

**Table 1:** Roles of Australian emergency responders.

Description of role	Number of people
Evacuation centre worker	4
Evacuation centre doctor	3
Family support recovery worker	3
Community family support worker	3
Early childhood educator	3
Midwife	2
Emergency researcher	2
Child services worker	2
Child friendly space worker	2
Social worker	1
Disaster welfare manager	1
Volunteers’ manager	1
Evacuation centre nurse	1
Donations manager	1
Recovery case worker	1
Independent volunteer manager	1
Health manager	1
Foster care manager	1
Family and child health nurse	1
Hospital-based nurse	1
Evacuation centre manager	1
Domestic violence worker	1
Disability support specialist	1
Catering manager	1
Total	39

**Table 2:** Country of operation of non-Australian emergency responders.

Country	Number*
Canada	3
Croatia	1
Iceland	1
Ireland	1
Italy	3
Japan	1
New Zealand	4
Poland	1
Ukraine	1
United Kingdom	2
United States of America	3
Total	20

^*^One participant had worked in two countries.

**Table 3:** Overview of themes and key findings.

Theme	Key findings
Challenges in supporting infants and young children in emergencies	
Lack of prioritisation, planning, and data collection regarding infants and young children	Infants and young children are not prioritised in emergency response and there are gaps in planning It is assumed parents do not need support caring for their children Data collection regarding infants and young children is inadequate
Lack of planning, knowledge, and appropriate support for IYCF-E	Vulnerability of infants and young children with regards to feeding in emergencies is not widely known It is falsely believed both that support is not needed for breastfeeding and that breastfeeding is fragile and will fail in an emergency
	Formula feeding is falsely believed to provide food security when compared to breastfeeding
	The support needs for formula feeding in an emergency are not appreciated and so are not provided for
Donations of infant formula	Donations of infant formula are made and are often excessive, out-of-date and uncontrolled in distribution Infant formula donations undermine appropriate IYCF-E practices
Interventions to support the mothers and caregivers of infants and young children	
Making support needs and gaps visible	Training of emergency workers and health professionals makes support needs visible Appropriate data collection, including needs assessments, help to identify gaps and required supports Involving those with expertise in infants and young children in disaster planning, preparedness and response enables identification of needs and appropriate response
Providing tailored evacuation centre support	Evacuation venues enabling families with infants and young children to be separated from the general population are beneficial Providing separate supported spaces in evacuation venues for families with young children enables the provision of timely and appropriate support, including for IYCF-E
Implementing IYCF-E interventions	Telephone-based breastfeeding counselling can provide effective support where in-person assistance is not available Policies and procedures on management of infant formula donations can reduce harm Educational resources on IYCF-E and other practical tools such as decision trees can help emergency responders to deliver appropriate infant feeding support
Supporting parents to provide responsive care in disaster recovery	Safe and supported spaces for mothers and caregivers are valued and can facilitate delivery of IYCF-E, health, and child development programs Public health campaigns can encourage parents to connect with and engage lovingly with their children
Facilitating intervention uptake	Training of staff and volunteers, support from community members, involvement of government agencies and UNICEF, and collaboration between health agencies and emergency organisations facilitate intervention uptake Government policy objectives towards IYCF-E planning can provide impetus for action UNICEF has a role in enabling support for infants and young children in emergencies even in high-resource settings
Promoting family resilience	Targeted engagement of parents with infants and young children can improve their individual preparedness Better overall community preparedness will mitigate against lack of individual family preparedness Strong support for breastfeeding pre-emergency will ensure better breastfeeding support during and after disasters

### 3.1 Challenges in supporting infants and young children in emergencies

The challenges experienced in supporting infants and young children in emergencies were largely consistent across the diverse countries in which study participants worked. They included (1) lack of prioritisation, planning, and data collection regarding infants and young children; (2) lack of planning, knowledge, and support for IYCF-E; and (3) the donations of infant formula.

#### 3.1.1 Lack of prioritisation, planning and data collection regarding infants and young children

Supporting the caregivers of infants and young children was experienced as a low priority in disaster planning and response.

Mothers and babies, they are definitely never a priority. It’s not something that people think about very much. [New Zealand].

I think emergency disaster management professionals do not think about infants and children and families. [Canada].

As a result, a planning gap for infants and young children in comparison to other areas of emergency response (including for animals) was noted.

They had protocols about removing artworks from the museum and storing them safely, or how to transport people from old age homes who could not walk … plans for animals … for what to do if there’s a lice breakout. I mean, they had all sorts of really detailed protocols about lots of different aspects of life, and yet nothing for pregnant women … and nothing for infants and young children. [Italy].

There’s nothing specific to children or infants … But they have [an action point] on … feeding and shelter for animals. [Ireland].

It was assumed parents would be able to look after their children without support, and this assumption was described as a possible reason for the lack of planning for infants and young children. An Australian emergency responder described a conversation she had with government disaster trainers,

One of the questions I sent back was, ‘So who looks after children under three? … Who gives the parents support?’ And when the question was asked, the three people that were facilitating it, just sort of looked at each other and said, ‘Oh the parents will know what to do.’

An Italian emergency responder summarised it as, ‘The idea is the mother knows.’

Emergency responders spoke of how lack of planning for young children meant mothers and caregivers must find their own solutions to challenges.

It’s like families figure it out, because they have no other option and people aren’t thinking about it. [USA].

The babies become an extension of the mother in a rest centre or in a disaster response, and whatever goes on … she’ll sort that. [UK].

Even in the well-resourced Ukraine War response, where some transit centres gave older children computer tablets to keep them occupied and happy, there was no provision to help a mother who was formula feeding reduce the risk to her infant.

[This mother] crossed the Ukrainian border … using very dirty bottles. I said, ‘How can this woman replace her bottle? She has three or four kids’ … [The response was], ‘She has to take a bus, she has to go to the city and find it out.’ I said, ‘So, there is no way we can support her? I mean we are supporting her with everything … The kids have laptops and tablets and I mean here there is no one that can help her? First help her clean her bottle in case she wants to clean what she has … and unfortunately, she’s using it … so we need to help her.’ No, the answer is, ‘She has to figure it out.’ Going back to that assumption that whenever it is about their babies, it’s her problem, she has to find a way. [Poland].

Emergency responders noted that older children appeared to be slightly better accounted for.

I think school [aged] children have been addressed … The infants are invisible, because they have mother and father, so what do they need more? [Italy].

Lack of consideration in data collection was also reported and likely contributed to the overall indiscernibility of infants and young children.

They know how many dogs and cats are there. You ask how many babies are there, they do not know. [Poland].

#### 3.1.2 Lack of planning, knowledge and appropriate support for IYCF-E

Lack of visibility of IYCF-E was noted as an issue needing addressing.

We did not recognise the very important need of mother to feed the baby. I do not know how it happened … This topic is invisible. [Poland].

This meant plans were not made to enable an appropriate response, even when required resources were available.

Anyone with an infant under the age of two months automatically got put into a hotel room, but they did not do any kind of assessment related to what their actual individual needs were. Is this baby breastfed? Then, that baby probably does not really need much other than access to nappies and maybe a change of clothes, right? But if this is a baby that is formula fed, they are putting them in hotel rooms without any [kitchen facilities] … They did not even think, ‘Oh, they might need a kitchenette.’ [Canada].

They had breastfeeding support groups very prepared and very strong and very rooted in the community. They were not allowed to enter the camps, because [disaster management] has no awareness. [Italy].

Lack of understanding that breastfeeding mothers might need support in emergencies contributed to lack of assistance.

Supporting breastfeeding and security in breastfeeding, it’s recognised as so natural … because mother produce the milk and everything is simple. [Poland].

However, the false belief that stress impacts breastmilk production was also widespread. This impeded provision of breastfeeding support and encouraged inappropriate infant formula distribution.

Some people believe that if you have a shock or if there’s some sort of big disruption to your life, like an earthquake, your milk supply will dry up no matter what you do … the idea was, ‘Okay, well if there’s been an earthquake, obviously you do not have any more milk so obviously you need formula.’ [Italy].

Breastfeeding was positioned as fragile while formula feeding was understood as providing food security.

People somehow have the idea that formula is … the food security and the breastfeeding somehow is going to fail. [Canada].

This resulted in planning and guidance focused on infant formula rather than breastfeeding.

The Government released the guideline to protect … pregnant women and mothers feeding [an] infant [in an] emergency … but it says that the breastmilk would stop so you need to carry the bottle and formula even if you are breastfeeding. [Japan].

Furthermore, while the OG-IFE recommends relactation and wet nursing, in Ireland it was believed the public would not be receptive to these practices, even though it was also recognised beliefs may change in an emergency.

As we are preparing and maybe delivering messaging around the possibility of some sort of large scale emergency, I think the idea of wet nursing and relactation will be generally unacceptable to our population. Now, I think that would change in the face of the emergency … I think conceptually people are like, ‘Oh no, wet nursing.’ But then if they have no access to food and their sister offers to feed the baby, I think they would change their mind pretty quickly and so I think it’s all very contextual.

Lack of appreciation of the vulnerability of infants related to feeding meant the urgency and importance of ensuring support was not well understood.

It is quite difficult to get breastfeeding on the table, and to get people to understand the complexities of it, and the impact … this person’s saying, ‘Oh, if the baby has not been fed for 18 hours, then we could do it tomorrow,’ that there’s just not that awareness at all. If you stop breastfeeding for three days, that’s actually quite a big thing, and difficult for people who do not have that in-depth knowledge to understand that this is not the same as what it would be for a five-year-old or an adult. [USA].

So, if someone was having a problem with even being able to breastfeed … when you come forward with a request, they are like, ‘That’s not on our priority list.’ And you are thinking, ‘Oh actually it should be. It’s got to be on someone’s priority list if this baby cannot feed.’ [Australia].

This lack of awareness extended to the resources needed for formula feeding in an emergency, and as a result this support was not appropriately provided.

But it was the same problem with formula because in the reception point there is the soap for the mother, there is the tea for the mother but there is not boiling water … to use with the infant formula. [Poland].

They were preparing formula … for their young infants … in [evacuation centre] bathrooms. [Canada].

We’d requested formula to be sent across, they sent one tin. We had a couple of hundred evacuees. One tin. We had multiple families dipping in and scooping out of this one tin, no sterilisation equipment … It was just an absolute health and safety nightmare. [Australia].

The complementary feeding needs of infants and very young children 6–24 months were also not understood and therefore not appropriately planned for or supported.

They had a feeding strategy for 0 to 5 and they said, ‘Well, they could eat pizza … 0 to 5 love pizza.’ And she’s like, ‘2 to 5 might love pizza, but there’s no feeding strategy for under 2.’ [UK].

They also did not have any kind of foods for children who were seven, eight, nine months old and starting solids. They had meals planned for older children, but no meal possibilities for younger children. [Italy].

They did not think, ‘Oh, this family’s going to need to prepare food for their children. They’re going to need the kitchen [in their evacuation accommodation].’ [Canada].

#### 3.1.3 Donations of infant formula

Infant formula donations were consistently a challenge across countries. These donations were often excessive, close to expiry or expired, and distributed in an uncontrolled way without ensuring caregiver access to other necessary resources.

You see visibly [there] is formula everywhere, all type of formula … tables and tables … that people can just grab and get it. Anyone can grab and get it … anyone. [Ukraine].

If you were a family arriving into a reception centre, you walked in and almost the very first thing you saw was a table full of formula … There was more formula than you could ever use here. There was no shortage of formula to begin with, and then it just flooded in. [Canada].

There was formula, and it was quite often just ‘take it if you want’, like it was just open slather … it always had a really short use by date. [Australia].

The formula, it was in the first kit provided in the train station, in the reception point … This formula was distributed completely uncontrolled. [Poland].

Emergency responders in several countries reported donations from the infant formula industry.

It was a huge mobilisation of the formula feeding company to provide plenty of them in the reception … they coming with a truck, with a lot of formula for refugee. [Poland].

There were absolutely crates and crates of formula that had been given. [New Zealand].

### 3.2 Interventions to support the mothers and caregivers of infants and young children

Interventions successfully implemented to improve support for the mothers and caregivers of infants and young children in emergencies varied between countries in terms of focus, nature of intervention, and resources invested. These included (1) making support needs and gaps visible, (2) providing tailored evacuation centre support, (3) implementing IYCF-E interventions, and (4) supporting parents to provide responsive care.

#### 3.2.1 Making support needs and gaps visible

Informal and formal training of emergency responders had been of benefit in several countries.

When you talk about it, when you sit down and discuss, they say, ‘Oh okay, we did not realise, we did not understand, we did not know.’ [Poland].

In non-emergency time, I made training courses for the coordinators of the Red Cross camps, starting from breastfeeding, why it is important? Because they really do not understand why we should care so much for breastfeeding when we have formula … it was useful for the awareness. But what works better is the case study. Prepare the case and tell them how you would manage these. [Italy].

In Italy, significant work had been undertaken to train health professionals working with new mothers and fathers on IYCF-E.

We included the infant feeding in emergency in the university curriculum of midwives in five universities. Which is great, because when they graduate as midwives, they know what it is. So, half of the job is done.

Involving professionals with infant and young child expertise in disaster planning, preparedness, and response was reported to facilitate the identification and consideration of issues affecting young children, ensuring these needs were brought forward and appropriately addressed.

We try to mix [the emergency organisations] with health system [people] … because we learned that not only multi-professional, but trans-disciplinary, trans-professional and trans-sectoral … has a transformative power for everyone. If the paediatrician or the midwife working at the community level works with Save the Children and with the community mother group, peer-to-peer groups and with the civil protection, they know each other and it’s very easy.’ [Italy].

Having someone there who knows about the resources and when a mission comes up, [says] ‘Hey, we have these kids that need to be fed,’ … that there’s somebody there with that broad base of education who says, ‘You know, I know a person or I know a group that we could call on to help us.’ [USA].

Recognising vulnerabilities associated with child age and feeding method was supported by appropriate data collection at evacuation centre registration and needs assessment at an individual and population level.

Needs assessments can be particularly helpful … that would include both a community needs assessment to first determine what’s already in place, and what’s needed to better support [caregivers] … in the community. But also conducting infant feeding needs assessments when families arrive at congregate or non-congregate shelter settings. [USA].

Emergency responders described the use of assessment tools.

We used some of the tools available through the Save the Children’s IYCF-E toolkit, adapted and modified some of those assessment tools. So, we were able to approximate the breastfeeding population that was impacted. [Canada].

We have that needs assessment in the toolkit, and we made it a fillable form so folks can download it … and then type in the information for the families and save it there so that they have it. [USA].

Finally, the involvement of individuals with international experience was identified as valuable in raising awareness of the particular vulnerabilities and support needs of caregivers of infants and young children in disasters.

Our project application [on supporting IYCF-E] came in and the [national] people had never seen anything like it before and they did not know what to make of it. They were on the verge of rejecting it when someone on the team said, ‘Maybe you should send that up to the international people.’ Then, it went up to the international people and they took a look at it and they are like, ‘Oh, yeah. It’s totally appropriate. There’s definitely a gap. [Canada].

#### 3.2.2 Providing tailored evacuation centre support

Emergency responders described interventions aimed at making evacuation venues more suitable for families with infants and young children. In New Zealand, the involvement of community members in the disaster planning process resulted in the deployment of an evacuation venue specifically for families with young children.

They had identified three community emergency hubs in their community. They sent one lot of people to the pub because they knew they were going to be rowdy … They sent families and small children to one of the Plunket [child health service] rooms and they sent the tourists … to one of the scout halls and then everyone else went to the rugby clubs. So, they were able to cater to the different groups.

In Iceland, schools are commonly utilised for evacuated populations and were described as facilitating the separation of families with infants and young children from the general evacuee population, thereby enabling the provision of better support.

If we have a parent with young children breastfeeding, we try to move them separately … most of our shelters are in schools, so it’s easy to have separate rooms.

Although not through deliberate planning, the value of multiroom venues as evacuation centres was also noted in Australia.

They had an evacuation centre in a large bowling club … [with] lots of areas for people to go to … and the families with young children … just naturally all went to the one area … I think that they had a much better experience.

In Alberta, Canada, a comprehensive intervention called the Baby and Child Unit was developed for families with children 0–3 years. The Baby and Child Unit was a separate supported space in evacuation venues which enabled assistance to commence immediately upon a family’s arrival.

They did not need to wait in line to be registered. They would be directed to the Baby and Child Unit’s area in their reception [evacuation] centre where they would then be registered in that unit. Their immediate needs and the needs of the children were taken care of first with feeding and hygiene a priority. Baby with dirty diaper, baby with no diaper, mother needing to feed immediately and needing a private space, people needing to make up bottles.

Within the Baby and Child Unit, mothers and other caregivers receive support and resources for:

- feeding (a private space for mothers to sit and breastfeed or express milk, snacks and water for breastfeeding mothers, infant formula, clean space for infant formula preparation, feeding implements, washing and sterilising supplies, access to water for washing, complementary foods)
- hygiene (space for nappy changing, nappies, nappy wipes, hand sanitiser)
- sleep (space for very young children to sleep including during the daytime, boxes for infants to sleep in, baby blankets)
- play (clean and contained space for infants and young children to crawl and sit and play, suitable clean toys)
- reassurance and emotional support provided by trained volunteers/workers.

Resource kits for Baby and Child Units are stored throughout the province, facilitating rapid deployment.

We created a whole kit that could easily be deployed that we had pre-stationed in each of our communities and several within the urban area … Signs that would go up in bathrooms, [stating] do not clean your baby feeding equipment here … diapers and wipes in every kit …. we had contact numbers of child feeding experts … and then we had things to keep the little siblings happy … colouring books and crayons, and little stuffed toys … so that the mother could at least have some time to feed while the little one was doing something else.

#### 3.2.3 Implementing IYCF-E interventions

While in-person breastfeeding counselling support may be ideal, enabling access to breastfeeding counselling by telephone had been of assistance in a number of countries. This included Japan where organisations collaborated to provide a telephone helpline service following the 2011 Great East Japan Earthquake.

La Leche League, the Japan Association of Lactation Consultants, and Breastfeeding Network … three organisations work together … with UNICEF to release information and build the free telephone counselling … ‘the UNICEF Baby Hotline.’

In Canada, evacuation centre workers connected mothers directly with pre-identified breastfeeding counsellors.

We could call them and say, ‘We have a mother here with twins … what do we do? [The breastfeeding counsellor speaks to the mother] and just you could hear and see the physical relief on the mother’s face as she was speaking … and the mother’s like, ‘Okay, we are going to be fine’.

Policies and procedures to prevent and manage donations of infant formula were described as very helpful.

The city … which ran the reception [evacuation] centre … had just brought in place a policy for procurement for all of their emergency responses, they had a new policy that they would accept no donated goods. And that was really helpful because it meant that we did not have to deal with donations of formula in that setting. [Canada].

In Australia, the USA, and Japan, flow charts were developed to help emergency responders to provide appropriate infant feeding support. These included questions for mothers and other caregivers and outlined referral pathways.

In Canada and the USA, a collection of educational resources on IYCF-E had been developed for both mothers/caregivers and health and emergency workers.

Posters, information around following safe feeding practices, what it means to prepare formula in an area where your water supply is not secure or it’s not clean. You cannot just still follow the recommendations on the back of the can anymore, you also have to combine that with your local medical officer’s recommendations for the water, things like that. We have materials available now across the zone. [Canada].

There’s handouts … in the toolkit for the emergency folks to then use with the families … and the idea, I think, too is that the materials would help educate the responders too. If they are going to be using them with families, then they would hopefully read the information. [USA].

#### 3.2.4 Supporting parents to provide responsive care to their children

In Australia, following the 2019–20 bushfires, a non-government children’s organisation implemented an intervention in a rural community called ChildSPACE to support the mental health, social connectedness and caregiving capacity of mothers and caregivers of young children. ChildSPACE provided a safe, comfortable and welcoming setting in which families with children could access emotional support, peer connection, health professionals and parenting support services, including structured activities such as baby massage classes.

Every time we are open we are just really quite busy with little people, and mums and grandmas come in. It’s just been such a place of connection … it’s just mum connecting with, or the carer connecting with, their baby … It’s about fostering connection and attachment.

In New Zealand, following the 2011 Christchurch Earthquake, a recovery program focused on encouraging parents to take time to engage lovingly with their children.

We did a thing called Tiny Adventures, and the byline was, ‘It only takes a minute to make a memory.’ It was literally a pack of cards, you could choose a one minute thing, a five minute thing, a 10 min thing, a half an hour thing … it was really simple, easy messaging … because what happened was that the caregivers were actually so busy focusing on everything else … these messaging were really simple and go, ‘Oh, actually, yes. I can spend five minutes’ … And it was a nice, simple messaging that everyone related to, and they could relate to … and they could still concentrate on getting the house secure, but also, in that little time with those zero to four children’.

### 3.3 Facilitating intervention uptake

Emergency responders described how intervention uptake was supported by training staff and volunteers, providing clear directions, piloting the intervention, and gathering the support of the local community. Each of these factors assisted the province-wide implementation of Canada’s Baby and Child Unit.

We did a pretend activation just for the Baby Unit. And we played it out with dolls … volunteers would come and pretend to be parents, grandparents, caregivers, and right down to putting a bracelet on the baby, a matching bracelet on the mother so that we knew who belonged to who … everybody knew what to do.

We gave them language that was very official. So it was, ‘You will put this poster at reception and you will ensure that you create a safe space, that is not a washroom, for … breastfeeding and you will direct people to it.

We also started [in one community] and then started teaching it to other communities in [Alberta] … So by the end of my three years there, we had just about every community trained.

It took quite a bit of negotiating with the municipality to really bring it in to be part of their plans, because it also required some budget on their end … The public health department up there worked really hard to establish very strong relationships with our indigenous communities surrounding the area and so we had a lot support … So along with us on the health side, the indigenous cultural pieces and the importance of family that they brought into the picture, I think just helped bring it all together.

Making sure the special needs of families with young children were understood by those with a broader focus also assisted in implementation of support measures that might otherwise have been perceived as unfair.

We would say things like, you are about to give out cash cards and there’s going to be eight-hour lineups, if you offer preferential access to people who show up who are mothers or people that are caregivers of very small babies, if you plan to give them preferential access, you will not interrupt their feeding, you will not cause further harm, and you also will not have so many crying babies in your lineup for hours and hours on end. [Canada].

Emergency responders in several countries described having government agency support as vital in gaining traction for interventions.

When a relevant government institution is on the lead, that’s very important, then you have a bit more traction around local governments … because you have a government institution behind it. [Italy].

Collaboration between health agencies and emergency organisations was a facilitator of uptake in Japan.

Good thing is we [health group] collaborate with the people from the field of emergency so that some of them very much … understand.

The inclusion of an action point to develop an IYCF-E policy in Croatia’s national breastfeeding strategy was successfully used to bring government and non-government organisations together to collaborate on developing IYCF-E planning based on the OG-IFE.

In our working group, we have a UNICEF representative, a representative of RODA [parent support organisation], we have a representative of the Ministry of Health, we have a representative of the nursing profession in Croatia, we have two representatives of the Ministry of Health who are members of our national breastfeeding committee, and … a representative of physicians.

Emergency responders from five countries described how the support of UNICEF had been of assistance in improving their emergency response to infants and young children, particularly regarding IYCF-E.

Their [UNICEF] involvement has been crucial, really. They’ve always been the ones supporting various IYCF-E initiatives … firstly, they are … an international body … Secondly, they have the respect and therefore the authority to do certain things. And thirdly, they have the organisational power … they know how to do these things … they have experience in the field. [Croatia].

### 3.4 Promoting family resilience

The importance of including parents, particularly mothers, in emergency preparedness activities was described by a number of emergency responders. Communicating via parenting and cultural groups had assisted with parental engagement in Canada.

We’d go and train mothers during their parenting groups. What are you going to do in case of an emergency? Well, here’s how it’s going to happen. So that when there was an evacuation, they have come, and they already know, ‘I need to go to the Baby and Child Unit.’ And they knew what to expect. And by doing that also eased a lot of the anxiety … And we found that going to the multicultural centres, the Islamic group, the East Indian Society, the Filipino Society, the newcomers … And we targeted those areas for training, ‘What do you need to put into your emergency kit if you have a baby? … And where do you keep this bag? And how often do you have to look at it?’

However, the demands of caring for very young children were also identified as a barrier to engagement. In New Zealand, an approach known as ‘community response planning’ was recognised as partly mitigating this challenge, reflecting the view that if the community as a whole was well prepared, families could be supported by others.

One thing that we find really difficult is actually targeting those families that do have young children because they are just so busy and they have got such other priorities. The last thing they want to do is think about emergency preparedness, which is where that community preparedness comes in. If a community is able to support themselves, well then, they know who’s in their community, what their community needs.

Similarly, it was also recognised that a strong health system that supported practices that improve resilience, such as breastfeeding, would be better placed to provide support in emergencies.

We are aware that if a system is protecting, supporting and protect breastfeeding in non-emergency time, they will do in emergency time in a perfect way. [Italy].

## 4 Discussion

This study revealed challenges and gaps in emergency planning and response to infants and young children across high-income countries as well as some successful interventions. Challenges and gaps appeared remarkably consistent despite diversity in geography, disaster type, culture, and healthcare and emergency management arrangements. A number of these challenges have been previously reported (13). Although disaster responses were well-resourced in these countries, the needs of infants and young children were reported to be broadly overlooked. This was not due to a complete lack of disaster planning, but rather because infants and young children were not prioritised, due in part to an underlying assumption that mothers would care for their children unaided. Illustrating this problem, study participants from Italy, Ireland, and Poland observed that there was better planning for animals than for infants. The same gap was previously identified in Australia and the UK. Thus, Australian government emergency plans were found to include the words ‘infant’, ‘baby’, or ‘babies’ 124 times whereas the words ‘animal’ and ‘pet’ were used more than 2,300 times (7). In the UK, emergency planning existed for pets, livestock, zoo and circus animals but the needs of mothers and infants were absent (14). The limited attention given to the needs of infants and their mothers and other caregivers in disaster planning seems largely responsible for many of the challenges emergency responders described.

Limited knowledge about IYCF-E, including breastfeeding and the requirements for safely supporting formula feeding in disasters, was evident in both planning gaps and, in some cases, the content of plans and guidance. For example, the Japanese guidance that advised breastfeeding mothers to have infant formula in case of a disaster, framed breastfeeding as fragile and unreliable, while positioning infant formula as a source of food security. This contrasts with evidence that disruptions to food supply chains, water and electricity, which often accompany disasters, can place formula-fed infants in a precarious situation, even in high-income countries (15). On the other hand, lactation and breastfeeding are robust physiological processes. As long as women remain hydrated and continue to provide regular breastfeeds to the infant, lactation can be maintained even in very difficult circumstances (16–18).

Breastfeeding is in fact the foundation of first food security (19) and it has been proposed that food security at a population level ‘is not possible without high rates of breastfeeding’ (20, p. 1). In emergencies, breastfeeding women can not only sustain their own infants but also provide a safety net for non-breastfed infants. Wet nursing and milk sharing in emergencies has been reported in high-income countries including in Australia, Croatia and Ukraine (21–24). Experiences of wet nursing and breastmilk sharing in besieged Mariupol in Ukraine in 2022 support this statement; women who breastfed and expressed breastmilk for multiple children were described as the ‘main source’ of nutritional security for infants during the siege (23). However, early communications regarding IYCF-E in Ukraine omitted wet nursing because it was considered culturally inappropriate (23). This aligns with the concern reported by this study’s Irish participant who also recognised that infant feeding risks may be misjudged and perceptions and practices around infant feeding may shift during emergencies, as was seen in Ukraine.

The assumption that mothers will be able to feed and care for their infants and young children in disasters without targeted assistance was described by study participants as contributing to the inadequate support for IYCF-E within formal emergency responses. This assumption reflects a lack of understanding of the needs of the youngest children, the important role of feeding support in ensuring their safety in disasters, and the complexities of IYCF-E in contexts where formula feeding is common. High-income countries experience high levels of partial or full formula feeding outside of emergencies (25). Additionally, infant feeding practices tend to deteriorate during emergencies rather than improve, likely fuelled by the commonly held belief that breastfeeding is prone to fail in a disaster (1). Research examining the experiences of Ukrainian mothers impacted by conflict in 2015 identified that nearly 60% of those who stopped breastfeeding their young infant did so because they believed stress or lack of food had reduced their milk supply (26). During the more recent conflict in Ukraine, women described healthcare providers encouraging them to formula feed their newborns because they believed breastfeeding was not possible in the context of war (27). Emergency responses that provide support for both breastfeeding women and those formula-feeding are needed in emergencies and recommended in the OG-IFE (28).

As outlined in the OG-IFE, the caregivers of infants and young children should be offered comprehensive support to mitigate risk (28). This should include the provision of breastfeeding counselling for breastfeeding women, the careful management of infant formula provision for non-breastfed infants, and complementary feeding support for children 6–24 months (28, 29). To protect both breastfed and non-breastfed infants, provision of infant formula should be tightly controlled and donations prevented (28). This is because donations of infant formula are invariably improperly distributed and jeopardise infant health including through reducing breastfeeding, unsafe preparation and feeding disruption resulting from an inconsistent supply (29). None of the countries in which study participants worked as emergency responders had plans based on the OG-IFE at the time of data collection. Participants reported multiple deficiencies in IYCF-E supports as well as challenges arising from uncontrolled donations and distributions of infant formula. Similar findings have been reported in other research (23, 27, 30–35). Hwang et al. (13) reviewed research on barriers and challenges to IYCF-E in middle- and high-income countries, identifying that across countries and in all emergency types, formula donations were problematic and in breach of the International Code of Marketing of Breastmilk Substitutes (36). With the exception of the USA, all countries represented in the research are members of the World Health Assembly and are therefore expected to have enacted legislation or regulations preventing the donation of infant formula in emergencies. To date, none have done so. However, since data collection, model policies and guidance for the management of infant formula donations, procurement, and distributions during emergencies has been developed for Australia and New Zealand to support organisational practice (37).

Of the countries represented, Croatia had taken the most substantive steps towards improving formal disaster planning, through a collaborative process to adapt the OG-IFE and integrate it into national planning. In late 2023, an IYCF-E plan was published by the Ministry of Health (38). This plan addresses each section of the OG-IFE, allocating responsibility to relevant agencies and providing resource contacts for emergency responders (39). This process and plan are major milestones for the implementation of the OG-IFE in high-income countries. A research project focused on IYCF-E on the island of Ireland (the Republic of Ireland and Northern Ireland) has published recommendations including, as a positive first step, the development of an IYCF-E plan (40). Whether this actually translates into action remains to be seen. In Australia, the 2019 government commitment to develop an IYCF-E plan (8) has yet to be actioned.

In terms of acute emergency response, the most substantial infant and young child-focussed intervention reported by study participants was the Canadian Baby and Child Unit. The wrap-around support to mothers and other caregivers of infants and young children within evacuation venues provided for IYCF-E practices aligned with the OG-IFE. The Baby and Child Unit could feasibly be adapted by other high-income countries to strengthen care for infants, young children and their caregivers during emergencies. Similarly, the Australian ChildSPACE intervention provided holistic care for mothers and other caregivers during disaster recovery, functioning as a type of Mother-Baby Area (MBA). The concept of the MBA was first developed during the Balkans conflict of the late 1990s and has been deployed in humanitarian disasters globally. While the specifics of MBAs vary according to context, their core functions are to provide pregnant women and mothers of infants and young children with a safe and private environment, opportunities for peer connection, access to health care and psychosocial support, and referral to appropriate services for other needs (41). Support for breastfeeding, formula feeding and complementary feeding are also provided via MBAs (42). The positive impact of MBAs was quantified in three refugee camps in Cameroon, where over 90% of the more than 1,000 women supported through MBAs demonstrated improved breastfeeding practices with reduced concern about insufficient milk supply, decreased maternal distress, and more positive mother-infant interactions (43). Similar positive effects were observed amongst mothers supported through MBAs in an Ethiopian refugee camp (44). Outside of humanitarian emergencies (conflict or refugee crises), it appears MBAs have not been widely used in disaster response in high-income countries. A guide for implementation of MBAs in Australia has been published and could be adapted for other high-income contexts (45).

Regarding implementation, interventions supporting infants and young children, training of emergency responders and health workers, involvement of health workers in disaster planning, and collaboration between health and emergency workers were reported as valuable in settings where these occurred. In Italy, ensuring relevant university curricula included content on IYCF-E is likely to have long-term benefit in building future workforce knowledge and strengthening emergency preparedness and response. Health workers have an important emergency response role in providing breastfeeding counselling and supporting safe preparation and feeding of infant formula and complementary foods. In fact, breastfeeding counselling is considered part of the minimum package of emergency health and nutrition services in disasters (46) and is referred to in the OG-IFE as an essential component of any IYCF-E response (28). Study participants described collaborations to provide breastfeeding counselling to mothers affected by disasters via off-site pre-identified breastfeeding counsellors or telephone helpline services. Telephone breastfeeding counselling has been shown to be effective in supporting women to breastfeed in non-emergency situations (47) and efficacy of video counselling was shown following an earthquake in Turkey (48). A recent innovation in Ukraine using artificial intelligence has enabled human-supervised digital support providing timely breastfeeding information with an ability to triage mothers requiring individual breastfeeding counselling (49). Other resources reported to aid implementation include signage, assessment forms, fact sheets and prompts for parents. A selection of resources and tools developed to support emergency response to infants and young children in the countries represented in this study can be found in Supplementary file 1. Finally, the involvement of a diverse range of stakeholders was reported to support effective implementation. This included government and non-government agencies, community members and UNICEF. Those with international humanitarian experience were noted as assisting with implementation. This is unsurprising, since supporting IYCF-E and the care of infants and young children are well established as vital components of humanitarian responses in low- and middle-income countries as described in the WHO’s nurturing care in emergencies brief (12).

Appropriate planning is considered the foundation for good emergency response, but planning alone is not enough. This study has demonstrated that planning and policies needed to be integrated across relevant government and non-government organisations to enable them to work together to protect very young children. The underrepresentation of pregnant women, parents and other carers of infants is common in emergency planning discussions, in part due to their caregiving responsibilities. An audit of the approximately 1,400 submissions made to an Australian government inquiry into natural disaster response following the 2019–20 bushfires saw just two submissions made by mothers of infants (9). Active inclusion of these groups and consideration of their perspectives is essential to ensure that plans and policies actually address their needs. The New Zealand community response planning approach may also be a way of ensuring that the needs of the youngest children and their caregivers are appropriately accounted for in disasters (50).

## 5 Strengths and limitations

It is a strength of this research that study participants were from diverse backgrounds; Australian interviewees held a broad range of emergency responder roles, while international interviewees were from a variety of countries with a diversity of disaster types and burdens. However, it is a limitation that the number of interviewees and countries represented was relatively few and some countries (Australia, New Zealand, Canada, the USA and Italy) were more heavily represented than others. This may limit the transferability of findings to countries with disparate approaches to emergency management. Furthermore, all interviews were conducted in English, which likely excluded some potential participants and may have restricted the depth of interview data obtained from participants for whom English was not their primary language.

Snowball sampling enabled key international informants with known relevant experience to be quickly invited to participate in the research. However, as a result individuals who may have had divergent relevant experience may have been overlooked. Nonetheless, the choice of sampling method was necessary and appropriate for the exploratory nature of the study and the short timeframe for data collection. The existence of a relationship between the interviewer and some of the study participants is both a strength and weakness, allowing for frank and easy conversation but also with the potential to restrict sharing of difficult experiences. Study participants were assured that anonymity would be maintained to assist in reducing this problem.

While data saturation was not sought or achieved, the concurrence of challenges described by study participants suggests that the most significant and impactful challenges may have been captured. However, not all positive interventions will have been described. Further research on IYCF-E and disaster response to infants and young children in high-income countries would be beneficial, including observational research.

## 6 Conclusion

Infants and young children are vulnerable in emergencies globally, including in high-income countries, and their mothers and other caregivers require IYCF-E and other support. However, lack of prioritisation, planning, and data collection has contributed to gaps in knowledge, training, resource allocation, and support for IYCF-E and the other needs of infants and young children in disasters in the high-income countries in this study. Addressing these gaps and challenges requires greater awareness, training, and integration of planning for infants and young children across all levels of emergency management. Some countries have begun adapting aspects of the OG-IFE and demonstrate that meaningful progress is achievable as well as providing valuable models for others to follow. Increased communication and collaboration between high-income countries has the potential to accelerate this process and should be encouraged.

## Data Availability

The datasets generated for this study are not available due to ethical reasons. Further inquiries can be directed to the corresponding author.
