# Supplementary material for: Support for infant and young child feeding and care in emergencies: experiences from Australia and eleven other high-income countries
Source: Front Nutr. 2026 Sep 17;13:1940793. doi: 10.3389/fnut.2026.1940793 (PMC13626898; doi:10.3389/fnut.2026.1940793)
Supplement: Supplementary file 1 [file Table_1.DOCX]

**Table S1.** Resources to support IYCF-E and other care of infants and young children in emergencies in high-income countries

| **Australia** |  |
| --- | --- |
| Australian Breastfeeding Association |  |
| https://www.breastfeeding.asn.au/information-emergency-workers-and-volunteers-supporting-families-emergencies | Orientation e-learning module on supporting families with infants and young children in emergencies |
|  | Emergency preparedness posters |
|  | Social media kit |
|  | Training exercise scenarios |
|  | Signage for evacuation and recovery venues |
|  | Evacuation centre evaluation tool |
|  | Supporting families in evacuation infographic |
|  | Supporting safer sleep for infants in evacuation settings |
|  | Model policies and guidance for managing infant formula donations, procurement and distribution in emergencies |
|  | Mother-baby area guide for Australia |
|  | Guide to supporting pregnant women and parents in disaster recovery venues |
| https://www.breastfeeding.asn.au/information-mums-and-families-feeding-and-caring-your-baby-emergency | Planning for emergencies tool and evacuation kit lists for parents of infants and young children |
|  | Fact sheets for mothers and caregivers on breastfeeding, expressed breastmilk feeding and formula feeding in emergencies |
|  | Fact sheets on cup feeding and hand expression in emergencies |
| **Canada** |  |
| Safely Fed Canada | Fact sheet for emergency responders on IYCF-E |
| https://safelyfed.ca/quick-reference/ | Signage for evacuation venues |
|  | Rapid infant feeding triage tool |
|  | Rapid infant feeding site assessment |
|  | Fact sheet on feeding infants in a sheltering situation |
|  | Fact sheet on making supportive spaces for parents |
| **Croatia** |  |
| Ministry of Health |  |
| https://zdravlje.gov.hr/prehrana-dojencadi-i-male-djece-u-kriznim-situacijama-smjernice-za-pruzatelje-pomoci-i-voditelje-programa-u-kriznim-situacijama/6105 | National IYCF-E guidelines for aid providers and programme managers in emergencies |
| **Italy** |  |
| UNICEF Italy |  |
| https://www.unicef.it/pubblicazioni/alimentazione-infantile-nella-emergenze-esercitazioni-per-testare-le-capacita/ | Infant feeding in emergencies training scenarios |
| National Institute for Health |  |
| https://www.epicentro.iss.it/allattamento/allattamento-durante-le-emergenze-2022 | Breastfeeding in emergencies |
| https://www.epicentro.iss.it/focus/terremoti/alimentazioneinfantile | Infant nutrition in emergences |
| https://www.epicentro.iss.it/ben/2018/settembre/alimentazione-infantile-emergenze | Six steps for implementing IYCF-E in line with the OG-IFE |
| **Japan** |  |
| Mother and Child Childcare Support Network |  |
| https://i-hahatoko.net/other-resources | IYCF-E assessment tree |
|  | Comic for mothers on infant feeding in emergencies |
|  | Booklet on preparing for disasters for breastfeeding women |
| https://i-hahatoko.net/our-resources/ | Checklist of precautions for liquid formula |
|  | Cup feeding in emergencies |
| Gender Equality Bureau Cabinet Office |  |
| https://www.gender.go.jp/policy/saigai/fukkou/pdf/guidelene_10.pdf | Breastfeeding assessment intake form and IYCF-E decision tree |
|  |  |
| Japan Breastfeeding Support Liaison Council |  |
| https://www.jlcobs.org/statement-201104/ | Joint statement on IYCF-E following the Great East Asian Earthquake |
| **New Zealand** |  |
| Canterbury District Health Board and the Mental Health Foundation of New Zealand |  |
| https://www.allright.org.nz/tools | Tiny adventures activities to connect parents and children |
| Health New Zealand |  |
| https://www.healthnz.govt.nz/health-topics/keeping-healthy/protecting-health-natural-disaster/during-an-emergency | Information for parents on infant feeding in a disaster |
| https://www.health.govt.nz/publications/infant-feeding-in-an-emergency | Position statement on infant feeding in emergencies |
|  | Emergency response roles and responsibilities on infant feeding |
|  | Guide for emergency responders on infant feeding |
| **United Kingdom** |  |
| Breastfeeding Network |  |
| https://www.breastfeedingnetwork.org.uk/breastfeeding-information/feeding-your-baby-in-an-emergency-situation/ | Information for parents on IYCF-E |
| **United States of America** |  |
| Centers for Disease Control and Prevention |  |
| https://www.cdc.gov/infant-feeding-emergencies-toolkit/php/index.html | Fact sheet on IYCF-E |
|  | Fact sheets on supporting infant feeding and creating family-friendly spaces in emergency shelters |
|  | Fact sheet on complementary feeding in emergencies |
|  | Rapid needs assessment for infant feeding |
|  | Rapid needs assessment intake form |
|  | Signage for evacuation venues |

The IYCF-E hub (https://iycfehub.org/) contains an extensive collection of resources developed for humanitarian emergencies, predominantly in low- and middle-income countries, that can be adapted for use in high-income countries.

The OG-IFE is published in languages including Arabic, Croatian, English, French, Italian, Japanese, Portuguese, Spanish and Ukrainian. It can be found at https://www.ennonline.net/resource/ife/operational-guidance-infant-feeding-emergencies-og-ife-version-30-oct-2017

Infographics summarising aspects of the OG-IFE are published in languages including Arabic, English, French, Italian, Japanese, Polish, Russian and Ukrainian.
